# Recognizing Hemophagocytic Lymphohistiocytosis (HLH) in an Urban Population: Clinical Characteristics, Treatment Patterns, and Outcomes

**DOI:** 10.1002/jha2.70360

**Published:** 2026-07-28

**Authors:** Safia Ansari, Kristy Bono, Merry Zhai, Aishwarya Subash, Ankit Shah, Joshua Kra

**Affiliations:** ^1^ Rutgers University New Jersey Medical School Newark New Jersey USA; ^2^ Rutgers Cancer Institute Newark New Jersey USA

**Keywords:** HLH, hemophagocytic lymphohistiocytosis, malignancy, urban, urban health, immunology, immunosuppression, HIV

## Abstract

**Introduction:**

Hemophagocytic lymphohistiocytosis (HLH) is a life‐threatening hyperinflammatory syndrome with high mortality in adults. Diagnosis remains challenging due to nonspecific clinical features and overlap with sepsis, malignancy, and other inflammatory conditions. Data on HLH in underserved populations are limited.

**Methods:**

We performed a retrospective cohort study of adult patients at a single urban tertiary care center from 2019–2025. Patients were identified using ICD‐10 codes for HLH and/or ferritin > 6000 ng/mL. Hscores and HLH‐2004 criteria were calculated to identify high‐probability cases. Patients were stratified into those diagnosed with HLH during hospitalization (Cohort 1) and those meeting criteria retrospectively without clinical recognition (Cohort 2).

**Results:**

Among 273 patients screened, 68 were identified as high‐probability of HLH, defined as an Hscore > 169 and/or fulfillment of at least five HLH‐2004 diagnostic criteria. Twenty‐five patients met inclusion criteria after secondary review. Seventeen patients were included in Cohort 1 and eight in Cohort 2. Cohort 1 patients were younger (median age 34 vs. 62 years) and predominantly Black or Hispanic, with high rates of uninsured status and social vulnerability. HIV (42.1%), infection (29.4%), and malignancy (17.6%) were the most common triggers. HLH‐directed therapy was administered in 82% of Cohort 1; mortality was 52.9%. In contrast, Cohort 2 patients demonstrated similar laboratory features but were not clinically recognized, with a high mortality rate of 87.57%. Hematology consultation occurred in 100% of Cohort 1 compared to 62.5% of Cohort 2.

**Conclusion:**

HLH is associated with high mortality and may be underrecognized in complex hospitalized patients, particularly older individuals with competing diagnoses. Early use of diagnostic tools such as the Hscore and prompt hematology consultation may improve recognition and outcomes, especially in high‐risk populations including those with HIV, malignancy, or severe infection.

**Trial Registration:**

The authors have confirmed clinical trial registration is not needed for this submission.

## 1 Introduction

Hemophagocytic Lymphohistiocytosis (HLH) is a rare, life‐threatening hyperinflammatory syndrome characterized by excessive, uncontrolled activation of macrophages and T cells causing inflammatory cytokine release and subsequent tissue injury [1, 2]. HLH can be classified into primary, which is caused by a genetic mutation, or secondary, which is acquired and occurs in association with underlying infection, malignancy, or autoimmune conditions [3]. Specifically, common triggers for secondary HLH include viral infections (herpes viruses, Epstein–Barr virus, human immunodeficiency virus, hepatitis B or C, cytomegalovirus), lymphoma, drug‐induced by bispecific antibodies, chimeric antigen T‐cell therapy, or checkpoint inhibitors, and systemic lupus erythematosus [4]. If left untreated, HLH often progresses to multi‐organ failure and death, making early identification and treatment critical for patient survival [2, 5, 6].

Despite standardized diagnostic criteria and emerging targeted therapies, mortality rates due to HLH remain high [4, 7]. Diagnosis is established by meeting the HLH‐94 or HLH‐2004 criteria or by calculating an Hscore. The HScore is a validated probability calculator developed for adults that estimates the likelihood of secondary HLH using readily available clinical and laboratory variables, including immunosuppression, fever, organomegaly, cytopenias, ferritin, triglycerides, fibrinogen, AST, and hemophagocytosis on bone marrow biopsy. An Hscore greater than 169 corresponds to approximately a 90% probability of HLH. The HLH‐2004 criteria also includes the additional values of low or absent natural killer cell (NK) activity and elevated soluble interleukin‐2 receptor (IL2R/CD25) activity [3, 6]. Despite these criteria, one major barrier to improved diagnostic capability is the significant variability and ambiguity in clinical presentation [8]. Typical clinical features and lab findings associated with HLH are often nonspecific in isolation and can mimic other inflammatory or infectious processes. The onset of symptoms may also be either gradual in nature or more drastic with rapid progression to shock [3, 5].

Recent guidelines, such as the National Health Service England's Getting it Right the First Time program, have sought to promote increased and earlier diagnosis by emphasizing the “3 Fs” as a clinical triad (fever, falling blood counts, and ferritin). This approach highlights the importance of considering HLH in patients with persistent fever, progressive cytopenias, and marked hyperferritinemia, particularly when the clinical course is disproportionate to the presumed underlying diagnosis [9, 10].

There is limited information regarding risk factors and demographic variables specific to HLH, especially among underserved patient populations in urban settings [11, 12]. In addition, specialized tests such as NK‐cell activity and IL‐2R levels may not be readily available in resource‐limited settings. Consequently, clinicians frequently rely on more readily available parameters, such as ferritin, cytopenias, and the HScore, to guide diagnostic and therapeutic decisions. A better understanding of these characteristics may enhance early recognition and increase the potential for targeted treatment options, ultimately improving patient outcomes.

We present a retrospective study on diagnosed and potentially undiagnosed HLH cases at a single Academic Medical Center located in a medically underserved, urban setting between 2019 and 2025. We aimed to identify demographic trends, clinical characteristics, and potential risk factors associated with HLH presentation to inform strategies for earlier recognition and improved management options in similar healthcare settings.

## 2 Methods

After obtaining Institutional Review Board (IRB) approval, adult patients ≥ 18 years old identified through two screening strategies: (1) ICD‐10 diagnosis codes for HLH (D76.1 and D76.2) and (2) serum ferritin levels >6000 ng/mL. Retrospective data was collected from electronic medical records (EMR) at a single tertiary care hospital. Data was extracted for all patients with an ICD‐10 diagnosis code of HLH (D76.1 or D76.2). In addition, patients with an elevated ferritin level > 6000 in the given time frame were included to capture cases of HLH that may not have been coded by ICD‐10 code. Ferritin level was chosen as a parameter since it is commonly drawn and levels above 6000 have been demonstrated at statistically significantly higher rates in patients with HLH compared to controls [13]. No additional medical records outside these screening criteria were reviewed.

Hscores and HLH‐2004 diagnostic criteria (see Table 1) were manually calculated for all patients, and patients who were considered high risk were identified. Based on the HLH‐2004 criteria, ≥ 5/8 of the diagnostic criteria are considered possibly positive for HLH [6]. The criteria were as follows: (1) fever; (2) splenomegaly; (3) cytopenia of at least two cell lines: hemoglobin <10 g/dL, platelets <100,000/µL, or absolute neutrophil count < 1000/µL; (4) elevated triglycerides level (>265 mg/dL) and/or low fibrinogen level (<150 mg/dL); (5) elevated ferritin levels >500 ng/mL; (6) elevated sIL‐2R levels (>2400 U/mL or more than two standard deviations from the mean value); (7) hemophagocytosis in the bone marrow, spleen, lymph nodes, or liver; (8) low/absent NK function. Similarly, an Hscore was calculated for each patient based on the following factors: known underlying immunosuppression, fever, organomegaly, cytopenias, ferritin, triglycerides, AST, and hemaophagocytosis features on bone marrow aspirate. Patients with an Hscore >169 and/or H‐2004 criteria >5 were further analyzed via secondary chart review.

**TABLE 1:** HLH‐2004 diagnostic criteria.

1. A molecular diagnosis consistent with HLH is made OR
2. Diagnostic criteria for HLH are fulfilled (five of the eight criteria below are fulfilled):
Fever
Splenomegaly
Cytopenias (affecting ≥ 2 of 3 linages in the peripheral blood: Hemoglobin < 90 g/L (in infants < 4 weeks: hemoglobin < 100 g/L), platelets < 100 × 10^9^/L, neutrophils < 1.0 × 10^9^/L
Hypertriglyceridemia and/or hypofibrinogenemia: (fasting triglycerides ≥ 3.0 mmol/L (i.e., ≥ 265 mg/dL), fibrinogen ≤ 1.5 g/L)
Hemophagocytosis in bone marrow, spleen, or lymph nodes
Low or absent NK‐cell activity (according to local laboratory reference values)
Ferritin ≥ 500 µg/L
Soluble CD25 (sIL2R) ≥ 2400 U/mL

## 3 Results

Cohort 1 consisted of patients who were diagnosed with HLH while inpatient, either through an ICD‐10 diagnosis code or by documented evaluation by a hematologist/oncologist. Cohort 2 consisted of patients who met criteria for HLH through retrospective chart review, but were not considered for HLH during their hospital stay.

A total of 273 patients who presented at our tertiary care center between 2019 and 2025 met initial inclusion criteria based on either an ICD‐10 diagnosis code or ferritin > 6000. Out of these 273 patients, 68 patients were identified as high risk for HLH (Hscore > 169 and/or H‐2004 criteria > 5). After thorough secondary chart review, the majority patients were excluded due low clinical suspicion for HLH based on other conditions (e.g. fulminant liver failure, sickle cell crisis) contributing to abnormal laboratory findings. Ultimately, 25 patients were included in the study.

### 3.1 Cohort 1

Seventeen patients were included in Cohort 1 (Tables 2 and 3). The median age of presentation was 34 years old. Ten patients were male (58.8%) and seven (42.1%) were female. Ten patients were Black (58.8%), five (29.4%) were Hispanic/Latino, and two (11.8%) were listed as Other race. Nine patients (52.9%) had no health insurance, six (35.3%) had Medicare/Medicaid, and two (11.8%) had commercial insurance. Three patients (17.6%) had a history of incarceration and two (11.8%) had documented food or housing insecurity.

**TABLE 2:** Cohort 1 demographics and clinical features.

Characteristic	*n* (%)
**Race**	
White	0 (0%)
Black/African American	10 (58.8%)
Hispanic/Latino	5 (29.4%)
Other	2 (11.8%)
**Sex**	
Male	10 (58.8%)
Female	7 (42.1%)
**Associated conditions**	
HIV+	7 (42.1%)
Infectious	5 (29.4%)
Malignancy	4 (17.6%)
Sickle cell disease	2 (11.8%)
Autoimmune	1 (5.9%)
Unknown	3 (17.6%)
**Insurance status**	
Commercial insurance	2 (11.8%)
Medicare/Medicaid	6 (35.3%)
No insurance	9 (52.9%)
**Social determinants of health**	
Incarceration	3 (17.6%)
Food/housing insecurity	2 (11.8%)
Substance use disorder	1 (5.9%)

**TABLE 3:** Cohort 1 treatments offered.

Treatment	*n* (%)
High dose steroids	11 (64.7%)
IVIG	6 (35.3%)
Etoposide	7 (35.3%)
Rituximab	4 (23.5%)
None	3 (17.6%)

In these patients, Hscores ranged from 156 to 246 and HLH‐2004 criteria ranged from 3 to 6. Ferritin level ranged from 10,763 to 125,888. Hematology/oncology was consulted in all of these cases (100%).

The mortality rate was 52.9%. There were a wide variety of underlying conditions that triggered HLH. Seven patients (42.1%) were HIV+, and five of them had a detectable viral load. Three patients (17.6%) had an active malignancy, including diffuse large B‐cell lymphoma and Hodgkin's lymphoma. Two patients (11.8%) had sickle cell disease, one patient had an autoimmune condition, and five patients (29.4%) had an active infection. Three patients (17.6%) had no clear trigger for HLH.

In terms of treatment, 82% of these patients received some type of HLH‐specific treatment. Six patients (35.3%) received intravenous immunoglobulin (IVIG), four (23.5%) patients received rituximab, and six patients (35.3%) received etoposide. Patients with lymphoma‐associated HLH were critically ill and did not receive definitive lymphoma‐directed therapy during the index hospitalization because of hemodynamic instability and multi‐organ dysfunction.

### 3.2 Cohort 2

Eight additional cases were identified that met predefined criteria for high‐probability HLH, defined as an Hscore >169 and/or fulfillment of at least five HLH‐2004 criteria, but HLH was not considered clinically during hospitalization. (Table 4).

**TABLE 4:** Cohort 2 demographics and clinical features.

Characteristic	*n* (%)
**Race**	
White	2 (25%)
Black/African American	4 (50%)
Hispanic/Latino	1 (25%)
Other	1 (25%)
**Sex**	
Male	5 (62.5%)
Female	3 (37.5%)
**Associated conditions**	
Malignancy	3 (37.5%)
HIV+	2 (25%)
Infectious	2 (25%)
Autoimmune	1 (12.5%)
Sickle cell disease	0 (0%)
**Insurance status**	
Private insurance	1 (12.5%)
Medicare/Medicaid	4 (50%)
No insurance	3 (37.5%)
**Social determinants of health**	
Incarceration	1 (12.5%)
Food/housing insecurity	1 (12.5%)
Substance use disorder	2 (25%)

The median age was 62 years old. Five patients (62.5%) were male and three (37.5%) were female. Four (50%) were Black, two (25%) were White, one (12.5%) was Hispanic/Latino, and one (12.5%) was listed as Other race. One patient was incarcerated, one had documented food or housing insecurity, and two had substance use disorders. Four (50%) had Medicare/Medicaid, three (37.5%) had no insurance, and one (12.5%) had commercial health insurance.

Hematology/oncology was consulted in 62.5% of these cases, often either for concerns of anemia or for a preexisting malignancy, but HLH was not documented as part of the differential diagnosis. Ferritin ranged from 9101 to 276,210. HLH‐2004 criteria ranged from 1 to 5, Hscores ranged from 175 to 221.

Three (37.5%) patients had an underlying malignancy, including cervical and breast cancer, two (25%) were HIV+, two (25%) had infection, one (12.5%) had an autoimmune condition.

The mortality rate in these patients was 87.5%. One of these patients received IVIG for treatment of another condition.

## 4 Discussion

HLH is highly lethal hyperinflammatory syndrome poorly described in an underserved, urban setting. In this single‐center study of patients presenting to an urban tertiary care center, we observed key unique features, including age and triggers for HLH. Importantly, there were demographic, clinical, and diagnostic differences between patients who were diagnosed with HLH during hospitalization and those in whom HLH met diagnostic criteria retrospectively but was not clinically considered. These findings underscore the ongoing challenges in managing this life‐threatening hyperinflammatory syndrome, particularly in medically and socially vulnerable populations.

In Cohort 1, where HLH was diagnosed during hospitalization, patients were relatively young with a median age of 34 years and were predominantly Black or Hispanic. These patients were younger than the reported average age of 49 years established in a literature review on adult HLH. More than half of these patients lacked health insurance, and several had documented social vulnerabilities including incarceration, housing insecurity, or food insecurity. Despite hematology/oncology consultation in all cases and HLH‐directed therapy in the majority of patients, mortality remained high at 52.9%. This mortality rate is consistent with previously reported adult HLH mortality rates ranging from approximately 40%–70%, mirroring the severe and rapidly progressive nature of the disease [4, 14].

Cohort 2 represented patients in whom HLH was not recognized during hospitalization and those in whom it was not considered. In these cases, HLH diagnostic criteria were met on retrospective review, yet the syndrome was not suspected clinically. Patients in Cohort 2 were older, with a median age of 62 years, and experienced markedly higher mortality at 87.5%, although this difference was not statistically significant, potentially due to the limited sample size. Several factors may explain this discrepancy. Older patients often present with multiple competing diagnoses, such as advanced malignancy or severe infection, which can obscure recognition of HLH. Laboratory abnormalities including cytopenias, liver dysfunction, and hyperferritinemia may be attributed to these conditions rather than prompting evaluation for HLH.

Current guidelines for the management of adult HLH emphasize early recognition and prompt initiation of immunochemotherapy tailored to the underlying trigger. Standard treatment strategies typically include corticosteroids and etoposide. These therapies aim to suppress the hyperinflammatory immune response by targeting activated T cells and cytokine production, with etoposide playing a particularly important role in controlling pathologic T‐cell proliferation. Additional options include cyclosporine and inhibitors of inertleukin‐1 (anakinra), interferon‐ γ (e.g., emapalumab), and Janus kinases (e.g., ruxolitinib) [3, 8]. Anakinra has been shown to reduce morality in patients with sepsis, and is recommended by the National Health Service for the treatment of primary or secondary HLH as a second line therapy [15, 16, 17]. IVIG and rituximab may be used in specific settings, including EBV‐driven disease. None of the patients in our cohorts received Anakinra, perhaps reflecting a lack of standardization in clinical practice. Given the heterogeneity of adult HLH presentations and frequent comorbidities, such as immunosuppression or infection, multidisciplinary care and early hematology/oncology involvement are strongly recommended to optimize treatment and mitigate complications [3, 8].

In this patient population, HLH triggers were heterogeneous, with HIV and active infections representing the most common underlying conditions. HIV was particularly prominent, with 42.1% of patients in Cohort 1 carrying a diagnosis of HIV and most demonstrating detectable viral loads (57%). HIV‐associated HLH may occur in the setting of uncontrolled viral replication, opportunistic infections, HIV‐associated malignancies such as Hodgkin lymphoma, or immune reconstitution inflammatory syndrome (IRIS) [12, 18]. Management of HLH in patients with HIV presents unique therapeutic challenges [19]. Treatment was differed in 17.6% of patients in Cohort 1, many of them HIV+, where there was concern that immunosuppression would lead to further clinical deterioration. In a meta‐analysis of 81 adult patients with HLH in HIV, 51% of patients received HLH‐directed therapy. In this cohort, 88% of patients were considered to have uncontrolled HIV, and survival rates were similar in controlled and uncontrolled HLH [12]. In our patient population, cases where HLH‐directed therapy was deferred include patients with viral loads > 1 million and infectious triggers such as disseminated histoplasmosis. There is no clear consensus on the optimal treatment for these patients—while guidelines recommend initiating dexamethasone and etoposide, in clinical practice, many patients are not provided HLH‐directed therapy. The role of these therapies are highly individualized and can vary based on infectious and noninfectious triggers [8, 12, 19]. This complex balance between immunosuppression and infection risk may contribute to treatment delays or modified therapy in HIV‐positive patients and likely plays a role in the high mortality observed in this population.

In addition, sickle cell crisis is a rare trigger for HLH, with only a limited number of cases described in the literature [20, 21, 22]. This may partly reflect the diagnostic difficulty created by overlapping clinical and laboratory features, including hyperferritinemia from repeated transfusions, cytopenias, and hepatosplenomegaly, which are commonly seen in both sickle cell crisis and HLH. In the cases of sickle cell–associated HLH observed in our cohort, infectious triggers such as influenza and cytomegalovirus were present. While cytopenias and systemic symptoms in typical sickle cell crises generally improve with supportive management, the persistence or progression of fevers, worsening cytopenias, and markedly elevated ferritin levels should raise concern for HLH. Additional biomarkers such as sIL‐2R levels may assist in differentiating HLH from severe inflammatory states; however, the time for these tests to result may limit their clinical utility in rapidly evolving disease.

About 40%–70% of HLH cases in adults are thought to be associated with malignancy, most frequently in T‐cell and NK‐cell lymphomas or leukemias, diffuse large B‐cell lymphomas (DLBCL), and Hodgkin lymphoma [8, 23, 24]. Malignancy was noted as an occasional trigger in our cohort, including both DLBCL and Hodgkin lymphoma. Malignancy‐associated HLH has consistently been associated with particularly poor outcomes in adult populations. The coexistence of malignancy, infection, and systemic inflammation often complicates both the diagnosis and management of HLH, as laboratory abnormalities such as cytopenias, elevated ferritin, and organ dysfunction may be attributed to the underlying malignancy or treatment rather than recognized as part of a hyperinflammatory syndrome. In addition, there is limited data on the treatment of HLH during chemotherapy, as patients are often profoundly cytopenic and immunsuppressed [23]. Although treatment of the underlying malignancy is generally recommended in malignancy‐associated HLH, the patients in our cohort were evaluated by a hematologist/oncologist and deemed too critically ill to receive definitive chemotherapy.

HLH remains a diagnostic challenge. The clinical triad of fever, bicytopenia, and splenomegaly should prompt further workup, but there is no single test that provides an unambiguous diagnosis of HLH, and genetic testing is generally not recommended in older adults as primary HLH would be exceedingly rare [2, 8]. Society guidelines recommend diagnosis based on meeting five out of eight diagnostic criteria; however, there are instances where HLH may be considered and HLH directed therapy may be initiated even when five criteria are not fulfilled. According to the guidelines, hyperferritinemia should always raise suspicion for HLH, although it is less specific for adults than it is for children [8]. In addition, there was a group of patients with elevated ferritin who had incomplete data, such as fibrinogen and triglyceride levels. Notably, 23 patients were at low or moderate risk and, if abnormal, these missing values could increase their risk to high risk. Thus, it often difficult to obtain a complete picture of all the high risk features. Importantly, one of the criteria includes hemphagocytosis on bone marrow, spleen, or lymph node biopsy. Only four patients included in our study underwent a diagnostic biopsy. Practically, the majority of these patients are critically ill, and obtaining a biopsy can be challenging, and often will not change management.

Differences in expert involvement may also have contributed to this diagnostic gap. Hematology‐oncology consultation occurred in all Cohort 1 cases but in only 62.5% of Cohort 2 cases. Early hematology involvement may facilitate diagnostic recognition and expedite treatment decisions in suspected HLH. Given the complexity of HLH diagnosis and management, multidisciplinary evaluation is often essential.

Our findings also highlight the potential influence of social determinants of health on HLH presentation and outcomes. A large proportion of patients lacked health insurance, and several had histories of incarceration, housing instability, or substance use disorders. These factors may contribute to delayed healthcare access, untreated infections, advanced immunosuppression, and more severe disease at presentation. Further investigation is needed to better understand how socioeconomic factors may influence disease recognition and outcomes.

Our study has several limitations. The sample size was small and limited to a single institution, which may limit generalizability. The retrospective nature of the study also introduces potential bias, particularly in determining clinical suspicion for HLH during hospitalization. In addition, some patients had overlapping conditions that may have complicated interpretation of laboratory findings. Despite these limitations, this study provides important insight into the clinical and demographic characteristics of HLH in an underserved urban population and highlights potential opportunities for improving early recognition.

## 5 Conclusion

Ultimately, our findings suggest that HLH may be underrecognized in complex medical patients, particularly older individuals with competing diagnoses such as malignancy or infection. HLH was associated with high mortality and frequently occurred in patients with underlying immunocompromising conditions and significant social vulnerability. The “3Fs” (fever, falling blood counts, and ferritin) may represent a simple and practical screening tool to prompt consideration of HLH, particularly in patients with competing diagnoses and limited access to specialized testing. HLH should be considered in patients with persistent fevers, cytopenias, and hyperferritinemia, particularly in cases of sepsis and multi‐organ failure not responding to treatment. HLH and sepsis can coexist, as sepsis can be a trigger for HLH. More widespread use of objective diagnostic tools such as the Hscore in patients with unexplained hyperferritinemia and systemic inflammatory features may help improve recognition. In addition, guidelines regarding HIV‐associated HLH rely on the fact that most cases of HIV are well controlled in the era of antiretroviral treatment, however this is not the case for our patient population with limited access to resources. There were several instances in the study where HLH‐directed treatment was deferred. More data is needed on optimizing treatment regimens in patients with HIV and other immunocompromising conditions. Finally, early hematology consultation and increased clinician awareness of HLH in high‐risk populations—including patients with HIV, malignancy, or severe infection—may facilitate earlier diagnosis and treatment.

## Author Contributions

**Safia Ansari**: conceptualization, methodology, data curation, formal analysis, investigation, writing – original draft, writing – review and editing. **Kristy Bono**: investigation, data curation, writing – review and editing. **Merry Zhai**: investigation, data curation, writing – review and editing. **Aishwarya Subash**: investigation, data curation, writing – review and editing. **Ankit Shah**: supervision, methodology, writing – review and editing. **Joshua Kra**: conceptualization, supervision, methodology, project administration, writing – review and editing. All authors have read and approved the final manuscript.

## Funding

The authors have nothing to report.

## Ethics Statement

This study was approved by the Rutgers New Jersey Medical School and University Hospital Institutional Review Board (IRB approval number: Pro2025000308).

## Consent

The requirement for informed consent was waived by the Institutional Review Board because this study involved retrospective review of existing medical records, posed no more than minimal risk to participants, and could not practicably be carried out without the waiver.

## Conflicts of Interest

The authors declare no conflicts of interest.

## Data Availability

The data that support the findings of this study are not publicly available due to the inclusion of protected health information and institutional restrictions. De‐identified data may be made available from the corresponding author upon reasonable request and with approval from the University Hospital Institutional Review Board.
